# The Impact of Early Cranial Doppler Ultrasonography on Prognosis in Neonates with Perinatal Asphyxia

**DOI:** 10.3390/children12060745

**Published:** 2025-06-09

**Authors:** Leyla Sero, Duygu Tuncel, Mehmet Salih Karaca, Nilufer Okur

**Affiliations:** 1 Clinics of Pediatrics, Gazi Yaşargil Training and Research Hospital, 21090 Diyarbakır, Turkey; 2 Clinics of Neonatology, Gazi Yaşargil Training and Research Hospital, 21090 Diyarbakır, Turkey; duygu.tuncel1@saglik.gov.tr (D.T.); nilufer.okur@saglik.gov.tr (N.O.); 3 Clinics of Radiology, Gazi Yaşargil Training and Research Hospital, 21090 Diyarbakır, Turkey; mehmetsalih.karaca@saglik.gov.tr

**Keywords:** neonatal, perinatal asphyxia, Doppler USG, resistive index

## Abstract

Background: Cranial Doppler ultrasonography (DS) is a non-invasive method for evaluating cerebral hemodynamics in neonates with perinatal asphyxia (PA). This study aimed to assess whether cerebral vascular resistance indices (RIs) measured within the first 24 h of life can predict the severity of brain injury. Methods: DS was performed on the anterior cerebral artery (ACA) and middle cerebral artery (MCA) between 6 and 24 h after birth in newborns diagnosed with PA. Prognostic value was evaluated by comparing RI values with cranial magnetic resonance imaging (MRI) results. Results: Of the 107 infants included in the study, 11 (10.3%) had severe brain damage, 27 (25.2%) had mild and 20 (18.7%) had moderate changes. The mean ACA RI was 0.61 ± 0.15 in the severe group and 0.70 ± 0.12 in the mild–moderate group (*p* = 0.023). MCA RI was 0.63 ± 0.20 and 0.71 ± 0.13, respectively. ROC analysis showed an area under the curve (AUC) of 0.901 for ACA RI with a cut-off of 0.58 (84% sensitivity and 84% specificity), and 0.874 for MCA RI with a cut-off of 0.59 (83% sensitivity and 84% specificity). Conclusions: Early ACA and MCA RI measurements via Doppler ultrasonography may serve as valuable predictors of brain injury severity in neonates with PA and should be considered alongside other clinical and imaging findings.

## 1. Introduction

Hypoxic–ischemic encephalopathy (HIE) is a serious neonatal brain injury caused by perinatal asphyxia (PA), and its early identification is critical for guiding treatment and predicting outcomes. The incidence of HIE varies depending on the diagnostic criteria, ranging from 2 to 9 per 1000 live births [1]. The only proven neuroprotective treatment is therapeutic hypothermia (TH), which is effective in reducing cerebral injury and improving outcomes when initiated within the first six hours of life [2,3]. Therefore, early and reliable prognosis is vital for the timely planning of interventions and for informing families and organizing long-term follow-up strategies.

Perinatal asphyxia leads to complex hemodynamic changes in the brain. In the early post-asphyxia period, cerebral blood flow is increased through reduced vascular resistance, elevated cardiac output, and systemic hypertension. However, the disruption of cerebral autoregulation and subsequent energy failure contribute significantly to the pathogenesis of brain injury [4]. Decreased cerebral blood flow (CBF), especially in the context of systemic hypotension, is a known risk factor for secondary ischemic injury [5,6,7]. Hence, monitoring cerebral hemodynamics provides valuable prognostic information.

Neuroimaging plays a central role in evaluating newborns with HIE. Magnetic resonance imaging (MRI), performed after therapeutic hypothermia (typically on days 3–5), is considered the gold standard for determining the extent of brain injury and long-term prognosis [8,9,10,11,12]. Cranial ultrasound (US) is commonly used in the early period to exclude structural abnormalities or hemorrhage. Findings such as increased echogenicity in white or gray matter and sulcal narrowing may indicate varying severity of brain injury. However, on its own, US lacks sufficient predictive power in the first days of life [8].

Doppler ultrasonography (DS), on the other hand, allows non-invasive bedside monitoring of cerebral blood flow patterns and vascular resistance. DS can reflect changes in cerebral hemodynamics, such as reduced flow due to increased intracranial pressure or decreased cardiac output [3,6,7,8]. Several studies have previously investigated the use of Doppler sonography (DS) and resistance index (RI) measurements. Archer [13] examined 43 term infants who exhibited signs of intrapartum asphyxia and survived the first two days of life. The anterior cerebral arteries of these infants were serially assessed using DS during hospitalization. In these asphyxiated infants, DS predicted outcomes with an overall accuracy of 86%. The sensitivity of abnormal Doppler findings in predicting adverse outcomes was 100%, and the specificity was 81%.

Similarly, Gray et al. [14] reported that abnormal mean blood flow velocity in the anterior cerebral artery (ACA) and low resistance indices in both cerebral arteries were significantly associated with poor outcomes in asphyxiated neonates. However, despite these promising findings, the number of comprehensive studies on this topic remains limited. Recent studies have explored the prognostic role of DS parameters like resistance index (RI) and pulsatility index (PI) in cerebral arteries. For example, Pishdad et al. [15] demonstrated that RI values from the middle cerebral artery (MCA), anterior cerebral artery (ACA), and basilar artery (BA) could help predict outcomes in asphyxiated newborns.

Furthermore, in the intrauterine period, the Doppler evaluation of MCA and ACA—particularly PI and peak systolic velocity—has been associated with adverse perinatal outcomes in fetuses with intrauterine growth restriction (IUGR) [16]. However, the literature on their postnatal prognostic utility in the setting of HIE remains limited, and further research is needed to determine the clinical significance of DS as a reliable and accessible tool for early prognostication. It is important to determine whether the relationship described above remains valid following the administration of therapeutic hypothermia.

Currently, in daily clinical practice, neurological prognosis in HIE is typically estimated using a combination of neuroimaging (MRI/US), amplitude-integrated EEG (aEEG), and clinical assessment [17]. While MRI remains the standard, accessible methods like cranial US and DS may serve as supportive tools, especially within the first 24 h, when decisions about hypothermia and adjunctive neuroprotective treatments must be made [18,19,20,21]. In this study, we aimed to determine whether early-stage DS measurements of RI in neonates with PA are a useful tool for predicting brain injury as seen on MRI.

## 2. Materials and Methods

This prospective study was conducted between 12 March 2023 and 15 April 2024 in a Level III neonatal intensive care unit. Neonates with a gestational age ≥ 36 weeks and diagnosed with PA were included in the study. Perinatal asphyxia was defined as the presence of one or more of the following conditions: umbilical cord blood or first-hour blood gas pH < 7.0 or base deficit (BE) < −12 mmol/L, Apgar score < 5 at 5 and 10 min, or need for intubation and/or cardiopulmonary resuscitation in the delivery room.

Therapeutic hypothermia was initiated in neonates who met the following criteria:

1. Demographic Criteria:
  ○ Gestational age ≥ 36 weeks;
  ○ Birth weight ≥ 2000 g;
  ○ Age ≤ 6 h at the time of assessment.
2. Biochemical Criteria:
  ○ Umbilical cord or blood gas pH ≤ 7.0, or base deficit ≤ −12 mmol/L within the first hour of life.
  ○ pH between 7.01 and 7.15, or base deficit between −10 and −15.9 mmol/L, accompanied by:
    ▪ An acute perinatal event (e.g., cord prolapse, placental abruption, uterine rupture);
    ▪ APGAR score ≤ 5 at 10 min;
    ▪ Need for assisted ventilation for ≥10 min after birth.
3. Neurological Criteria:
  ○ Moderate or severe encephalopathy, as defined by the Sarnat staging system, or presence of seizures on amplitude-integrated EEG (aEEG).

Infants with incomplete information, patients transferred to another hospital before MRI, or patients who died during hospitalization before MRI were excluded from the study.

Ethical approval was obtained from the local ethics committee (25 February 2022; no: 34).

## 3. Protocol and Patient Evaluation

Infants with a history of acute perinatal events and umbilical cord blood gas values suitable for the study were evaluated by a neonatologist. AEEG monitoring was initiated. Sarnat scoring was performed, and infants were grouped into mild (Stage 1), moderate (Stage 2), and severe (Stage 3) asphyxia [22]. Infants in the moderate and severe encephalopathy groups were treated with whole-body therapeutic hypothermia to maintain a body temperature between 33.5 and 34.5 °C. Therapeutic hypothermia was maintained for 72 h, followed by gradual rewarming using a servo-controlled device to raise body temperature to 36.5–37 °C over 8 h.

All infants underwent routine bedside ultrasound examination 6 to 24 h after birth on the first day of life. Brain MRI and diffusion MRI were performed between the 4th and 7th days following the completion of hypothermia therapy.

On the first day of life, cranial ultrasound (US) imaging was performed by an experienced radiologist, and DS, MCA, and ACA RI and PI measurements were taken and recorded. Measurements were obtained from the proximal branches of the anterior cerebral artery and the right middle cerebral artery. Doppler ultrasound imaging was performed using a 12 MHz linear probe (Voluson P6, General Electric, Boston, MA, USA) in all patients.

Previous studies incorporating Doppler ultrasound (DS) RI measurements have used different cutoff values. In one study, normal RI values were accepted as 0.56–0.89 [15]. RI values above and below these values were considered abnormal. The pulsatility index is an ultrasound flow parameter obtained from the maximum, minimum, and average Doppler frequency shifts during a defined cardiac cycle. Together with the resistance index, it is frequently used to assess resistance in pulsatile vasculature.

At the end of therapeutic hypothermia treatment, cranial and diffusion MRI scans were performed when the infants were 4 to 7 days old. The MRI images were interpreted by another radiologist who was not aware of the US results. The severity of hypoxia was assessed based on cranial and diffusion MRI scans performed between the 4th and 7th days of life.

Weeke et al. [23] reported that assessments of deep gray matter, white matter, and the cerebellum are associated with neurodevelopmental prognosis in the first two years of life. In T2 sequences, deep gray matter involvement (basal ganglia, thalamus, internal capsule, brainstem, hippocampus, perirolandic cortex) was classified as severe HIE. A limited hyperintense T2 signal in the cerebral cortex and subcortical white matter represented mild HIE, while the presence of more widespread cortical, cerebral white matter, optic radiation, corpus callosum, and punctate white matter lesions was defined as moderate HIE.

The patients included in our study were divided into two groups based on cranial MRI findings: One group consisted of patients with severe MRI findings. The other group consisted of infants with mild or moderate MRI findings or normal MRI findings.

These two groups were first compared based on ACA and MCA RI and PI values in DS. Subsequently, the cutoff points, specificity, and sensitivity of these values were determined for severe asphyxia. The relationship between measurements in DS and adverse findings in cranial US and EEG was also evaluated.

During measurement, comfort was provided to the patients using methods such as midline position, flexion position, loose swaddling, and pacifiers for non-intubated patients.

### Statistical Analysis

Data were analyzed using Statistical Package for Social Sciences for Windows software (SPSS Inc., Chicago, IL, USA), version 22. Descriptive data were presented as *n*, with % values for categorical data.

The X^2^ test was used to compare categorical variables. When comparing categorical variables, Fisher’s Exact Test was applied if the number of observations was less than 5. The Shapiro–Wilk test was used to check whether the arithmetic data were normally distributed. For data showing a normal distribution, the mean ± standard deviation was used; for data not showing a normal distribution, the median (minimum–maximum) was used. The Mann–Whitney U test was used for variables that did not show a normal distribution. Due to the small sample size, data are expressed as median (minimum-maximum). The Spearman correlation test was used for variables that did not show a normal distribution. Receiver Operator Characteristic (ROC) analysis was used to determine the cutoff points of RI and PI measured by Doppler US for MCA and ACA and to perform specificity and sensitivity analyses. Statistical significance was accepted at *p* < 0.05 in the analyses.

## 4. Results

A total of 112 newborns were included in the study. Five (4.5%) patients died before undergoing cranial MRI during follow-up. Data from a total of 107 patients were included in the study. According to the Sarnat staging system, 30 (28%) infants were classified as Stage 1, 42 (39.3%) as Stage 2, and 35 (32.7%) as Stage 3. Therapeutic hypothermia was administered to 77 (72%) patients.

When demographic data were analyzed, the groups showed similar characteristics in terms of birth weight, maternal age, and gender. Patients in Sarnat Stage 3 had a higher likelihood of being born via normal spontaneous vaginal delivery. Infants of mothers with maternal risk factors for acute perinatal events such as placental abruption had a higher likelihood of developing Stage 3 PA. The 5 min APGAR score was lower in Stage 3 compared to Stages 1 and 2 (*p* < 0.01 and *p* < 0.01). In umbilical cord blood gas analysis, pH levels were lower in Stage 3 and Stage 2 than in Stage 1, with no difference between Stage 3 and Stage 2. BE levels, however, differed across all HIE stages. The demographic and perinatal characteristics of patients according to Sarnat scoring stages and comparisons between stages are presented in Table 1.

Patients were classified as having normal, mild, moderate, or severe involvement based on cranial MRI findings. Eleven (10.3%) infants had severe involvement and 96 (89.7%) had normal, mild, or moderate involvement. Of the MRIs, 49 (45.8%) were normal, 27 (25.2%) showed mild involvement, and 20 (18.7%) showed moderate involvement.

Pathological findings were detected in 21 patients on cranial US: increased echogenicity in 15 patients, bleeding in 4 patients, and brain edema with sulcal effacement in 2 patients.

In cranial MRI, pathological US findings were present in 7 (78%) patients in the severely affected group and in 14 (14.3%) patients in the mild–moderate group (*p* < 0.01).

ACA-RI was 0.64 ± 0.098 in patients with pathological cranial US findings and 0.7 ± 0.12 in those with normal US findings, and a statistically significant difference was found (*p* = 0.038).

In 30 (28%) of the patients, antiepileptic treatment was initiated due to clinical and subclinical seizures during follow-up. Among patients receiving antiepileptic treatment, 17 (56.7%) had pathological US findings, which was higher than in those without antiepileptic requirement (*n* = 77, pathological US findings in 4 [5.2%]) (<0.01).

According to Doppler US measurements of RI, the mean ACA RI was 0.61 ± 0.15 in the severe PA group and 0.7 ± 0.12 in the mild–moderate PA group, with a statistically significant difference observed (*p* = 0.023). MCA RI was 0.63 ± 0.2 in the severe PA group and 0.71 ± 0.13 in the mild–moderate PA group (*p* = 0.048). There were no differences in ACA and MCA PI measurements between the severe PA and mild–moderate PA groups (*p* = 0.29 and *p* = 0.46, respectively) (Table 2).

**Figure 1:**
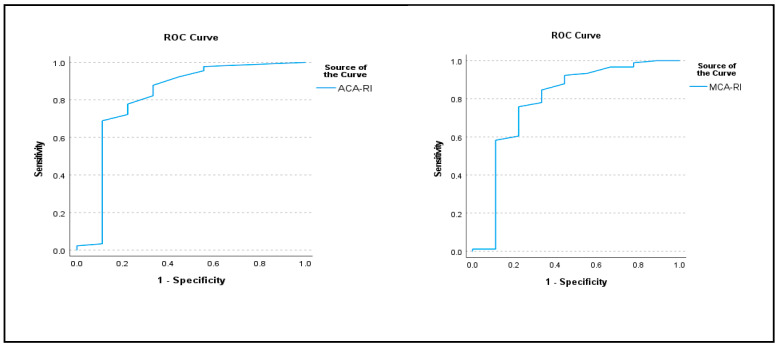
ROC analysis of ACA RI and MCA RI.

When ROC analysis was performed, the AUC value for ACA RI was 0.901 and the cutoff point was 0.58; sensitivity was 84% and specificity was 84%. For MCA RI, the AUC was 0.874 and the cutoff point was 0.59; sensitivity was 83% and specificity was 84% (*p* = 0.01 and 0.01, respectively) (Table 3, Figure 1).

During follow-up, a total of 5 (4.7%) patients died. ACA RI and MCA RI were evaluated between deceased and surviving patients. The ACA RI level was lower in deceased patients compared to surviving patients (mean 0.59 ± 0.07 and 0.69 ± 0.11, respectively, *p* = 0.032). MCA RI, ACA PI, and MCA PI measurements were similar (*p* = 0.11, 0.35, 0.42, respectively).

## 5. Discussion

In this prospective study, we found that ACA and MCA RI Doppler US measurements performed on the first day of life may help better assess the prognosis of term and preterm newborns with perinatal asphyxia.

The implementation of therapeutic hypothermia as a neuroprotective treatment in patients with moderate to severe encephalopathy has improved prognosis. This approach has facilitated the prompt identification of infants presenting with perinatal asphyxia who are at the highest risk for developing moderate to severe HIE. The early recognition of candidates who may benefit from current and emerging neuroprotective interventions enables more efficient resource allocation and supports timely prognostic assessment. Current indicators such as blood biochemistry, clinical examination, and electrophysiology are limited and their predictive power is reduced by the effect of therapeutic hypothermia intervention; however, they remain central to our predictive tools in the critical first hours after birth [24]. Additionally, higher-than-expected disability rates have been reported following therapeutic hypothermia treatment in mild HIE cases [25]. This highlights the need for additional tools to predict prognosis.

Monitoring changes in cerebral blood flow following perinatal asphyxia may be a useful option for assessing prognosis. Doppler ultrasound is a safe, non-invasive, and repeatable bedside imaging method that can help us understand neonatal cerebral hemodynamics [26]. There is evidence that cerebral blood flow is selectively maintained for vital brain stem structures during asphyxia. As systemic circulation returns, cerebral blood flow exceeds pre-ischemic levels. In an experimental study, cortical blood flow in dogs remained at at least 20% of the normal levels for at least 18 h after acute asphyxia. Post-asphyxia cerebral hypoperfusion is caused by arteriolar spasm. Previous reports have shown that RI values measured by DS remain low for 24 h or even days after asphyxia [13].

Allison et al. [27] found that the mean ± standard deviation of ACA and MCA RI levels in newborn infants on the first day of life was 0.726 ± 0.057.

Recently, a few studies have measured DS RI levels, particularly those indicating short-term prognosis. Pishdad and colleagues [15] evaluated a total of 34 patients using Doppler US in a multicenter cross-sectional study. They found that Doppler measurements of the ACA, MCA, and basilar arteries could be correlated with MRI findings (r > 0.8 and *p* < 0.001). They reported that ACA and BA RI values ≤ 0.62 were significant for distinguishing between normal and asphyctic patients, with 95% sensitivity and 100% specificity.

In a case–control study conducted by Jongeling and colleagues [28], RI values below 0.55 were associated with poor prognosis and had a positive predictive value of 71%.

In a study by Kudrevieiene et al. [29], ACA RI was measured between the 1st and 5th days of life. The study showed that ACA RI ≤ 0.55 measured between the 1st and 5th days was associated with damage to the thalamus, basal ganglia, and cerebellum.

In our study, we obtained results similar to those found in the existing literature. The threshold values were 0.58 for ACA RI and 0.59 for MCA RI. However, while higher specificity and sensitivity values were reported in the literature, our values were 84% and 84%, respectively. These values indicate the severity of the lesion on cranial MRI. We could have obtained better results if we had included patients who died before MRI. However, although the deceased patients formed a small group, we found a correlation with ACA RI values, but MCA RI values did not differ between the mortality group. DS measurement, although somewhat difficult, especially in term infants, was performed by an experienced radiologist who had no prior knowledge of the patients’ conditions. Therefore, we can conclude that our measurements were adequate.

Guan and colleagues reported that ultrasound findings and cerebral blood flow parameters are useful in the early diagnosis of HIE and help predict the severity of the disease and neurodevelopmental [8] outcomes. Similarly, our study showed that early ultrasound findings and parameters indicating cerebral blood flow, as measured by DS, may be useful in assessing the prognosis of HIE.

In a study by Cizmeci and colleagues, it was reported that ultrasound features and cerebral blood flow parameters are useful in the early diagnosis of HIE and assist in predicting neurodevelopmental outcomes. Cranial US may play a complementary role as the first neuroimaging method for detecting hypoxic–ischemic injury, but brain MRI remains necessary to confirm neurodevelopmental prognosis [30]. In line with the results of the previous study, our current study found that severe pathological ultrasound findings were present in the severe HIE group, as determined by cranial MRI results (*p* < 0.01).

Proietti and colleagues reported that seizure activity observed on EEG in newborns with HIE indicated moderate to severe injury [30]. Sae Yun and colleagues reported that clinical seizures in infants with HIE were independently associated with abnormal neurodevelopment [31].

In this study, patients with pathological ultrasound findings had a higher incidence of severe MRI findings and antiepileptic use. Cranial ultrasound, including Doppler measurements, is necessary for evaluating and monitoring neonatal HIE, provides information about cerebral hemodynamics, and aids in prognosis. Using Doppler ultrasound, clinicians can assess cerebral blood flow by measuring parameters such as RI and PI. Abnormal RI values indicate severe HIE and are associated with poor neurodevelopmental outcomes. Additionally, early abnormalities observed on cranial ultrasound, such as changes in brain parenchymal echogenicity and ventricular dilatation, may serve as predictors of long-term neurodevelopmental impairments. Therefore, combining cranial ultrasound findings with clinical assessments such as the Sarnat score and amplitude EEG improves the accuracy of outcome prediction in newborns with HIE [8,30,31,32].

Early determination of the prognosis in perinatal asphyxia can significantly influence treatment decisions and improve outcomes. Early diagnosis of at-risk infants enables the prompt initiation of treatment and reduces the severity of brain damage. Early prognosis assessment helps clinicians select more appropriate candidates for this treatment. Infants with severe asphyxia and poor prognosis may require intensive support in a neonatal intensive care unit that offers advanced neuro-monitoring, ventilation, and neurology consultations. In contrast, infants with mild symptoms may avoid unnecessary aggressive interventions. Early prognostic information allows for informative and compassionate discussions between healthcare teams and families about potential outcomes. Long-term planning for follow-up and early intervention services (such as physical therapy, occupational therapy, and speech therapy) can be initiated earlier. Infants diagnosed with moderate to severe encephalopathy in the early stages can be enrolled in neurodevelopmental follow-up programs. The early diagnosis of high-risk infants allows for closer monitoring of cerebral palsy, cognitive delays, or epilepsy symptoms. Early prognosis determination enables better patient selection for neuroprotective studies, thereby improving research quality and potentially leading to the development of new treatments [24,33].

Our study has some limitations. Some factors that could affect Doppler US ACA and MCA RI levels (such as hypothermia treatment) were not considered. We did not have a control group of healthy infants. Finally, our study does not include long-term neurodevelopmental outcomes.

## 6. Conclusions

Perinatal asphyxia can lead to various neurodevelopmental outcomes, with the timing of these outcomes varying across different developmental stages. Early diagnosis and intervention are important to address the neurodevelopmental challenges associated with perinatal asphyxia. In this way, early physiotherapy and rehabilitation are planned to minimize developmental delays in future children. Monitoring and support throughout infancy and early childhood are crucial for optimizing developmental outcomes.

Ultrasound findings, including ventricular size, brain echogenicity, and Doppler measurements (ACA and MCA RI), are helpful in predicting neurodevelopmental outcomes. While MRI remains the gold standard, early DS performed within the first 24 h, especially in severe cases, provides valuable additional information.

## Figures and Tables

**Table 1:** Characteristics of study patients.

	Sarnat Stage 1 (*n*= 30)	Sarnat Stage 2 (*n* = 42)	Sarnat Stage 3 (*n* = 35)	*p*
Birth weight, g *	3165 ± 357	3233 ± 474	2973 ± 625	0.15
Gestational age, week *	37.8 ± 0.94	38.2 ± 1.4	37.2 ± 2.3	0.12
Male, *n* (%)	16 (52)	24 (57)	28 (80)	>0.05
C/S, *n* (%) • Stage 1 × Stage 2 • Stage 1 × Stage 3 • Stage 2 × Stage 3	24 (80)	32 (76.2)	17 (48.5)	
				0.56
				0.03
				<0.01
Pre-eclampsia, *n* (%)	1 (3.3)	0	3 (8.6)	>0.05
Maternal diabetes, *n* (%)	4 (13)	2 (4.8)	4 (11.5)	>0.05
Cord entanglement *n* (%)	2 (6.6)	2 (4.8)	4 (11.5)	>0.05
Placental abruption *n* (%) • Stage 1 × Stage 2 • Stage 1 × Stage 3 • Stage 2 × Stage 3	2 (6.6)	3 (7.1)	6 (17)	
				0.12
				0.02
				<0.01
Meconium delivery *n* (%)	10 (33)	9 (21.4)	12 (35)	>0.05
Uterine rupture, *n* (%)	0	0	2 (5.7)	>0.05
Shoulder dystocia, *n* (%)	1 (3.3)	2 (4.8)	2 (5.7)	>0.05
Maternal COVID *n* (%)	2 (6.6)	2 (4.8)	3 (8.6)	>0.05
5th min APGAR ** • Stage 1 × Stage 2 • Stage 1 × Stage 3 • Stage 2 × Stage 3	6 (5–8)	6 (4–7)	4 (3–6)	
				0.53
				<0.01
				<0.01
Umbilical cord pH * • Stage 1 × Stage 2 • Stage 1 × Stage 3 • Stage 2 × Stage 3	7.05 ± 0.1	6.98 ± 0.12	6.92 ± 0.18	
				0.014
				<0.01
				0.1
Umbilical cord BE * • Stage 1 × Stage 2 • Stage 1 × Stage 3 • Stage 2 × Stage 3	−13.6 ± −2	−16 ± −2	−19 ± −4.3	
				0.027
				<0.01
				0.038

* Mean ± std. deviation; ** median (minimum-maximum).

**Table 2:** Comparison of cranial Doppler USG measurements according to severity of perinatal asphyxia.

	Severe PA (*n* = 11)	Mild–Moderate PA (*n* = 96)	*p*
Resistive index *			
ACA	0.61 ± 0.15	0.7 ± 0.12	0.023
MCA	0.63 ± 0.2	0.71 ± 0.13	0.048
Pulsatility index *			
ACA	1.63 ± 0.5	1.2 ± 0.3	0.29
MCA	2.5 ± 1.1	1.1 ± 0.4	0.46

* Mean ± std. deviation.

**Table 3:** Cut-off value of predictors of adverse outcome.

	AUC	Cut-Off Value	Sensitivity (%)	Specificity (%)	*p*
Resistive index					
ACA	0.901	0.58	84	84	<0.01
MCA	0.874	0.59	83	84	<0.01

## Data Availability

Datasets analyzed or generated during this study are presented in the current study.
